# Study of Sex Differences in Duloxetine Efficacy for Depression in Transgenic Mouse Models

**DOI:** 10.3389/fncel.2017.00344

**Published:** 2017-10-31

**Authors:** Yong Xu, Lei Ma, Wei Jiang, Yuhong Li, Gang Wang, Rena Li

**Affiliations:** ^1^The National Clinical Research Center for Mental Disorders & Beijing Key Laboratory of Mental Disorders, Beijing Anding Hospital, Capital Medical University, Beijing, China; ^2^School of Life Sciences, University of Science and Technology of China, Hefei, China; ^3^Beijing Institute for Brain Disorders, Capital Medical University, Beijing, China; ^4^Center for Hormone Advanced Science and Education, Roskamp Institute, Sarasota, FL, United States

**Keywords:** sex difference, duloxetine, depressants, estrogen, mice

## Abstract

Clinical evidences show sex differences in risk of developing depressive disorders as well as effect of antidepressants in depression treatment. However, whether such a sex-dependent risk of depression and efficacy of antidepressants is dependent on endogenous estrogen level remain elusive. The aim of this study is to explore the molecular mechanisms of sex differences in antidepressant duloxetine. In the present study, we used genetic knockout or overexpression estrogen-synthesizing enzyme aromatase (Ar) gene as models for endogenous estrogen deficiency and elevation endogenous estrogen, respectively, to examine the anti-depressive efficacy of duloxetine in males and females by force swimming test (FST). We also measured the sex-specific effect of duloxetine on dopamine and serotonin (5-HT) metabolisms in frontal cortex and hippocampus (HPC). Elevation of brain endogenous estrogen in male and female mice showed a reduction of immobility time in FST compared to control mice. Estrogen deficiency in females showed poor response to duloxetine treatment compared to sex-matched wildtype (WT) or aromatase transgenic mice. In contrast, male mice with estrogen deficiency showed same anti-depressive response to duloxetine treatments as aromatase transgenic mice. Our data showed that the sex different effect of endogenous estrogen on duloxetine-induced anti-depressive behavioral change is associated with brain region-specific changes of dopamine (DA) and 5-HT system. Endogenous estrogen exerts antidepressant effects in both males and females. Lacking of endogenous estrogen reduced antidepressive effect of duloxetine in females only. The endogenous estrogen level alters 5-HT system in female mainly, while both DA and 5-HT metabolisms were regulated by endogenous estrogen levels after duloxetine administration.

## Introduction

Major depressive disorder (MDD) is reported to be one of the most common mental health challenges in the world (Mitchell et al., 2011). It is known that depression is affected by sex, age and hormonal status in human and animal studies. Women have higher prevalence of MDD than men in general, but the differences fainted away slowly in aged populations (Hyde et al., 2008; Solomon and Herman, 2009). While the mechanism of sex differences in MDD remains unclear, one of the major hypotheses is that females are more sensitive in the hypothalamic-pituitary-adrenal axis (HPA) related hormones than males (DeSantis et al., 2011). For example, depression in women are associated with an increased sensitivity to changes in the hormonal milieu, such as the luteal phase of cycles, the postpartum period and during the menopause transition (Young et al., 2000; Maartens et al., 2002; Payne, 2003). Some research showed that female with low level of estrogen is associated with the higher risk of MDD (Young et al., 2000; Maartens et al., 2002; Payne, 2003), and estrogen treatment can treat depression in perimenopausal women, improve the happiness of menopause women (Schneider et al., 1997; Schmidt, 2005a,b), alleviate depressive symptoms in females (Sherwin, 1994), and manage hormonal-related depression in females (Soares, 2014, 2017). Lacking of estrogen in animals also cause significantly increase in immobility and less swimming in force swimming test (FST) and reverse the depressive behavior in FST (Imwalle et al., 2005; Vega Rivera et al., 2016). While estrogenic functions in regulating behavioral states such as mood and cognition have been relatively well documented in both human and animal studies, the effectiveness of estrogen therapy in depression is still remained controversial as some clinical studies show no effects of estrogen replacement therapy on reversing depressive-like symptoms for postmenopausal women (Arnold et al., 2004; Morrison et al., 2004; Goldstein et al., 2005; Pefanco et al., 2007; Martel et al., 2009).

Dysregulation within central monoaminergic systems has been believed to be a major underlie the pathology of depression. During the past decades, dopamine (DA), norepinephrine (NE) and serotonin (5-HT) have been the major targets of antidepressants (Zocchi et al., 2003; Elhwuegi, 2004; Andrews et al., 2015). For example, reduction of monoaminergic function has been improved by anti-depressant treatment, suggesting a connection between modified monoamine metabolism and depression (Papakostas et al., 2007; Antkiewicz-Michaluk et al., 2017). The two major classes of antidepressants used to treat MDD are the selective serotonin reuptake inhibitors (SSRIs), and serotonin–norepinephrine reuptake inhibitors (SNRIs; Linde et al., 2015). Compared to SSRI, SNRI antidepressant is a relatively new class of antidepressants that affect both 5-HT and NE uptake while both neurotransmitters are known to help regulate mood (Maron and Shlik, 2006). Some studies found a more potent effect of SNRIs on the 5-HT system than the NE system (Rueter et al., 1998; Béïque et al., 1999; Rénéric et al., 2002).

While evidence indicates sex difference in MDD, some previous reports suggest that women respond to antidepressant treatment for MDD differently from men. Fluvoxamine (SSRIs) treatments were more effective in younger women than older women (Morishita and Arita, 2003). There was no significant difference between premenopausal and postmenopausal women in treatment response to imipramine, a tricyclic antidepressants (TCAs; Vermeiden et al., 2010). Some studies reported a tendency of better or worse effect of SNRI for MDD in female patients than that in male patients (Morishita and Arita, 2003) as well as in animal studies (Xing et al., 2013), many other clinical studies demonstrated no sex differences in antidepressant effects of SNRIs treatments on MDD patients (Thase et al., 2005; Stewart et al., 2006; Naito et al., 2007). However, while some studies have suggested sex differences in the efficacy of SNRI antidepressant medications, there have been few investigations into potential sex differences in SNRIs efficacy in MDD treatment is regulated by endogenous estrogen levels.

Duloxetine is a SNRI antidepressant and showed sex-specific effects on treatment for MDD related conditions. For example, duloxetine treatment in female MDD patients demonstrated greater improvement with overall sexual function compared with male patients (Hudson et al., 2007), improved fibromyalgia symptoms and pain severity in female subjects, not in males (Arnold et al., 2004) and induced more dry mouth and fatigue in females (Brunton et al., 2010). Plus, duloxetine has been sued for treatment of aromatase inhibitor-associated musculoskeletal symptoms in women (Henry et al., 2011). Although duloxetine efficacy for MDD does not show significance between male and females, the sex-specific effect of duloxetine on other condition suggest a potential linkage between the antidepressant and female hormones.

Estrogens as the primary female sex hormone can be synthesized by aromatase, a key enzyme responsible for converting androgen into estrogens. Aromatase can be found in both males and females in various tissues including gonads, adipose tissue, blood vessels, skin, bone and brain (Callard et al., 1977, 1978; Steimer and Hutchison, 1980; Payne and Hales, 2004; Cui et al., 2013). Mouse with genetic aromatase gene knockout lacks the ability to synthesize estrogens *in vivo* and have been used as an estrogen-null model for study interaction between endogenous estrogen and disease pathologies as well as various drug-induced actions (Yue et al., 2005; McAllister et al., 2010; Kurokawa et al., 2015). In contrast, aromatase transgenic mice increase estrogen synthesis and can be a model for endogenous estrogen enrichment to investigate the effect of elevated endogenous estrogen on depressive behaviors and interaction with antidepressants.

The present study was designed to assess whether the efficacy of duloxetine treatment in male and females is regulated by endogenous estrogen and the possible underlying mechanisms of the sex-difference in duloxetine-induced antidepressive action in mice. Two genetic animal models were used for estrogen deficiency (genetic knockout aromatase, Ar^+/−^) and enhanced brain estrogen synthesis (genetic overexpression of neuronal specific aromatase, Thy1-Ar), respectively, while wildtype (WT) mice as model for normal level estrogen as we previously described (Yue et al., 2005). Compared to ovariectomized animal model for estrogen deficiency, these genetic animal models are more suitable for studying endogenous estrogen dependency brain function (Prange-Kiel and Rune, 2006), especially it is known that brain is able to synthesize estrogen from cholesterol independent from circling estrogen (Do Rego et al., 2009). Animals were tested for depressive-liked behavior by FST followed by measurement of DA and 5-HT turnover in the various brain regions in responding to duloxetine treatment.

## Materials and Methods

### Animals

All mice were maintained in accordance with National Institutes of Health Guide for the Care and Use of Laboratory Animals and with the approval of the IACUC in the Roskamp Institute. The aromatase gene knockout (Ar^−/−^) female mice with C57Bl/6J genetic background were generated by target disruption of exons1 and 2 of the Cyp19 gene as previously described (Honda et al., 1998). Heterozygous mice of Ar^+/−^ were generated by breeding male Ar^−/−^ mouse with female WT mouse. The brain specific aromatase transgenic mouse model (Thy1-Ar) was generated by Thy-1.2 gene with C57Bl/6J genetic background. Neuron specific expression of human aromatase gene was modified by Thy1.2 genomic expression cassette. Both Ar^−/−^ and Thy1-Ar mice were maintained by crossing with F1 breeders having a C57Bl/6J background. Litter-matched WT were used as control animals. Mice were housed four per cage in standard plastic cages with bedding and were maintained on a 12:12 h light–dark cycle (lights on at 08:00) with access food and water *ad libitum*. Mice were moved into the behavioral test room at least 1 h before the experiment. Before behavioral experiments, mice were divided into two groups, as duloxetine treatment and vehicle treatment. At age of 2–3 months, mice in duloxetine group received 10 mg/kg duloxetine intraperitoneally (i.p.) 30 min before the FST and open-field test (OFT), while vehicle mice received distilled water (10 ml/kg) injection. There were 6–7 mice each treatment group. The effective dose of duloxetine (10 mg/kg) were chosen based on previous publications (Santana-Coelho et al., 2016; Xue et al., 2017). Behavioral experiments were conducted between 21:00 and 24:00 to minimize circadian influence.

### Genotyping

The mice were genotyped using PCR. Tail tissue was digested with Proteinase K overnight at 56°C, and genomic DNA was isolated using a DNeasy Tissue Kit (Qiagen, Valencia, CA, USA) and amplified by PCR using the following primer pairs: pair 1: 5′-AGCCCTCAAGGTAAATGGGGA-3 and 5′-GAGGATGTGCCCTCATAATTCC-3′, for Thy1-Ar; pair 2: 5′-CCTTGACGATCGTTCATAC-3′ and 5′-GAG AGTTCATGAGAGTCTGG-3′, for the aromatase mutated gene (Honda et al., 1998; Bakker et al., 2002). PCR was carried out at the following parameters: 94°C, 1 min; 65°C, 2 min; 72°C, 3 min; 35 cycles. The PCR products were separated by 1% agarose gel electrophoresis and detected by staining with ethidium bromide.

### Drug

Duloxetine (Sigma, USA) were dissolved in distilled water before the test.

### Forced Swim Test (FST)

The FST experimental procedure was conducted as described previously with minor modifications (Ji et al., 2014). Briefly, after the single injection of duloxetine or vehicle, mice were individually tested for FST for 6 min in a glass cylinder (14 cm diameter, 25 cm height), containing water at level of 15 cm with 24 ± 2°C. The fresh water was refilled between trials in order to keep the same water level. The immobility duration of mouse movement was recorded during the last 4 min of the 6-min testing period. The immobile time of each mouse were identified as the mouse floated in the water without struggling. The experimental procedure was recorded by digital video-camera. The immobile time was scored by individuals who were blind to animal genotype information. At the end of the FST, mice were sacrificed by decapitation. Brain tissue as well as blood sample were harvested immediately and stored at −80 until assay.

### Spontaneous Locomotor Activity

Spontaneous locomotor activity was measured in mice by an OFT performed as described previously with slight modifications (Mutlu et al., 2017), in order to ensure that the changes in immobility of mice were not due to alterations in locomotor activity. In brief, each mouse was placed gently on the center square of a 50 × 50 × 50 cm plastic box after 30 min after vehicle or duloxetine treatment and its distance moved were recorded by a digital video-camera for 5 min and then data were analyzed by Ethovision software version 8 (Noldus, Netherlands). The apparatus was cleaned with 10% ethanol after each trial.

### High Performance Liquid Chromatography (HPLC)

Mouse brain tissue were dissected and homogenized in 0.2 ml ice-cold 0.4 M perchloric acid on ice followed by centrifugation at 12,000× *g* for 20 min, 4°C. The supernatants were then mixed with a buffer (2:1 in volume) contains 20 mM Potassium citrate, 300 mM Dipotassium hydrogen phosphate and 2 mM EDTA and incubated on ice for 1 h in dark followed by another centrifugation (12,000× *g*, 20 min, 4°C). Then the supernatants were filtered through 0.22 mm cellulose filters (Millipore, USA). Twenty microliter of filtered samples were injected into a high performance liquid chromatography (HPLC) system (Model 5600A; CoulArray Detector System, ESA, Chelmsford, MA, USA). The contenting of DA, DOPA, HVA, 5-HT and 5-HIAA in the prefrontal cortex (PFC) and hippocampus (HPC) were measured by HPLC combined with electrochemical detection (HPLC-ECD; Jia et al., 2017). The results were presented as ng/g protein by conversion. DA and 5-HT turnover rates were indicated by DOPAC+ HVA/DA and 5-HIAA/5-HT ratios, respectively (Muneoka et al., 2009; Del Pino et al., 2017).

## Statistical Analysis

All data was presented as the means ± SEM and evaluated by *T*-test or one-way analysis of variance (ANOVA). Statistical significance was considered at *P* < 0.05. The SPSS version 20 for Windows (SPSS, IBM, USA) was used to analyze the data.

## Results

### Overexpression of Aromatase Reduces Immobility Time in the FST

**Figure 1:**
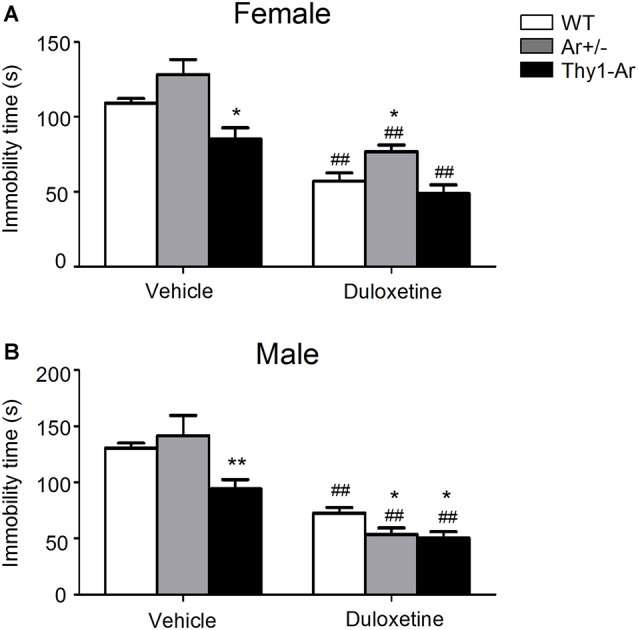
Immobility time in the force swimming test (FST) of three genotype in both female and male mice treated by duloxetine. The immobility time in the FST of three genotype in female mice **(A)** and male mice **(B)** treated by duloxetine. ^##^Indicates *P* < 0.01 compared to vehicle-treated, *indicate *P* < 0.05 and **P < 0.01 compared to wildtype (WT) mice mice. *n* = 6–7 mice/group.

To investigate the effect of endogenous estrogen on depressive behavior, we examined vehicle treated Ar^+/−^ as an estrogen deficiency model and Thy1-Ar as a brain estrogen overexpression model while age- and sex-matched WT mice as controls for FST. At age of 2–3 months, vehicle treated female Thy1-Ar mice spent less immobile time than that of WT mice, while a similar effect of brain estrogen on FST in male Thy1-Ar mice was also observed (Figures 1A,B). Both male and female Ar^+/−^ mice with endogenous estrogen deficiency showed a trend of increasing depressive behavior as measured by immobility compared to sex-matched WT mice, while the changes did not reach statistical significance. Data suggested that overexpression of brain estrogen might have an anti-depressive effect on FST in both male and female Thy1-Ar mice compared to sex-matched WT mice.

### Duloxetine Treatment Reduced Depressive Behavior in Both Male and Female Mice in all Three Genotype

To study effect of SNRI on depressive behaviors in male and female mice, a single dose of duloxetine (10 mg/kg, i.p.) was administrated to WT, Ar^+/−^ and Thy1-Ar mice minutes prior FST. As shown in Figure 1A, female mice treated with duloxetine showed significant reduction of immobile time compared to vehicle treatment regardless genotypes. A similar effect of duloxetine on FST was also found in male mice in all three genotypes as shown in Figure 1B. When we compare the effect of duloxetine on different genotypes in females, our data showed no significant change between female Ar^+/−^ and female Thy1-Ar and WT, although a trend of increasing immobile time was found in female Ar^+/−^ mice (Figure 1A). However, male Ar^+/−^ mice treated with duloxetine showed less depressant-like behavior than that of male WT mice (Figure 1B).

### Duloxetine Do Not Affect Locomotor Activity in Both Male and Female Mice in all Three Genotypes

**Figure 2:**
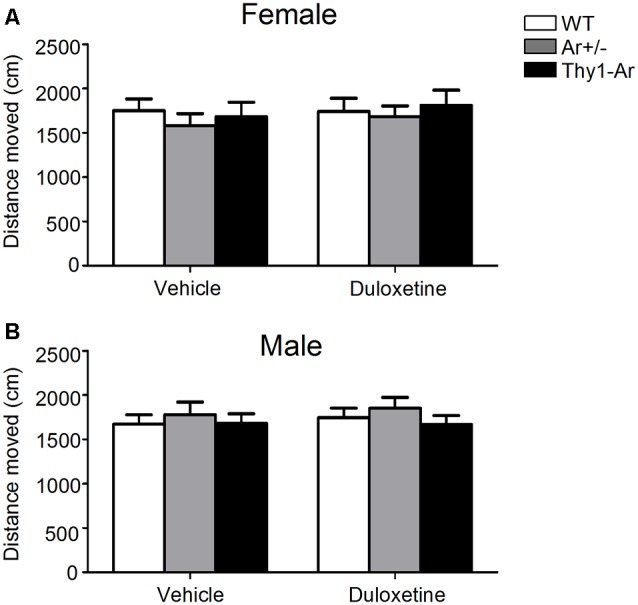
Duloxetine do not affect locomotor activity in both male and female mice in all three genotypes. The distance moved in the spontaneous locomotor activity of three genotype in female mice **(A)** and male mice **(B)** treated by duloxetine showed no differences in all three genotype treated by vehicle or duloxetine. *n* = 6–7 mice/group.

In order to determine whether duloxetine affected general motor activity of different endogenous estrogen level in both male and female mice, we tested spontaneous locomotor activity of mice with all three genotypes treated by duloxetine. As shown in Figure 2, there was no significant effect of endogenous estrogen in total distance moved regardless of sex difference. Furthermore, administration of duloxetine (10 mg/kg) failed to change the distance moved in genotype—matched mice of both male and female.

### Sex Differences in Effect of Endogenous Estrogen on DA and 5-HT Index in the PFC and HPC

**Figure 3:**
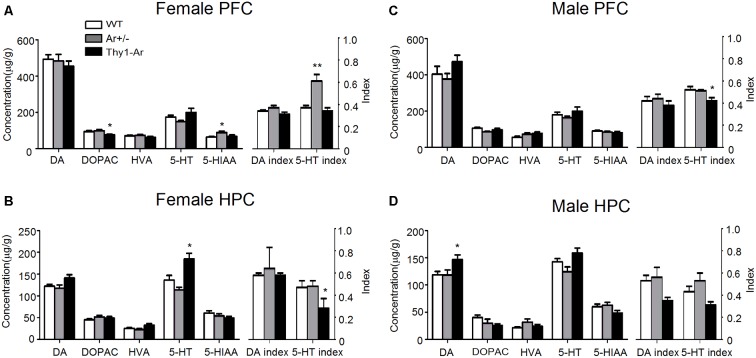
Sex differences in the effect of endogenous estrogen on dopamine (DA) and serotonin (5-HT) turnover rates in the prefrontal cortex (PFC) and hippocampus (HPC). The effect of endogenous estrogen on DA and 5-HT index of three genotype in female PFC **(A)**, female HPC **(B)**, male PFC **(C)** and male HPC **(D)**. *Indicate *P* < 0.05 and **P < 0.01 compared to WT mice. *n* = 6–7 mice/group.

To better understand the effect of endogenous estrogen on SNRI-induced anti-depressive action in behaviors, we measured DA and 5-HT with their metabolites by HPLC analyses in the PFC and HPC of the experimental mice. For DA and its metabolites, we found reduction of DOPAC level in the PFC of female Thy1-Ar mice compared with that of female WT as shown in Figure 3A, while no changes of DA and its metabolites were found in the HPC in all three genotypes female mice (Figure 3B). Elevation of DA level was noticed in the HPC of male Thy1-Ar mice without any changes in the PFC in the three genotypes male mice (Figures 3C,D).

In contrast to DA metabolism, there were elevated 5-HIAA level and 5-HT index with no changes in level of 5-HT in the PFC of female Ar^+/−^ mice (Figure 3A). No change of 5-HT and its metabolites in the HPC of female Ar^+/−^ mice compared to female WT mice (Figure 3B). Compared to WT, female Thy1-Ar mice showed an increased 5-HT level and reduction of 5-HT index in the HPC, with no change in the PFC compared to WT (Figures 3A,B). The effect of estrogen on 5-HT metabolisms is sex dependent. As shown in Figures 3C,D, compared to WT, male Thy1-Ar mice showed no differences of 5-HT and 5-HIAA levels regardless of brain regions, except a significant reduction of 5-HT index in the PFC.

### Sex Difference in Effect of Duloxetine on DA and 5-HT Index in PFC and HPC

**Figure 4:**
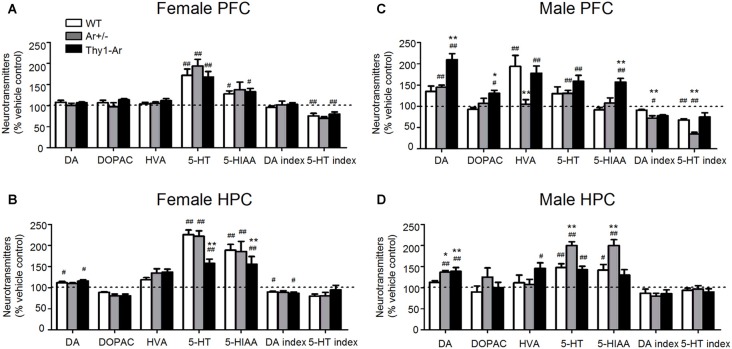
Sex differences in the effect of duloxetine on DA and 5-HT index in the PFC and HPC. The effect of duloxetine on DA and 5-HT index of three genotype in female PFC **(A)**, female HPC **(B)**, male PFC **(C)** and male HPC **(D)**. ^#^Indicates *P* < 0.05 and ^##^*P* < 0.01 compared to vehicle-treated, *indicate *P* < 0.05 and **P < 0.01 compared to WT mice. *n* = 6–7 mice/group.

Duloxetine administration promotes DA levels in the brain of male mice more than that in female mice, particularly in the PFC of male Thy1-Ar mice compared to male WT and Ar^+/−^ mice (Figures 4A,C). For example, female WT, Ar^+/−^ and Thy1-Ar mice treated with duloxetine showed no differences in DA and its metabolisms in the PFC compared to vehicle treatment, while elevated levels of DA and its metabolites were found in males with genotype and brain region dependency (Figures 4C,D). Duloxetine treatment caused an increase of DA level and reduction of DA index in the HPC of female WT and Thy1-Ar mice compared to vehicle treatment while no significant changes found in the female Ar^+/−^ mice (Figure 4B). Interestingly, male Thy1-Ar mice showed greater responses in the levels of DA and its metabolites in the PFC than male WT and male Ar^+/−^ mice, except HVA level, while male Ar^+/−^ mice showed no changes of HVA level compared to vehicle treatment (Figure 4C). In the HPC, duloxetine elevated DA levels in the male Ar^+/−^ and Thy1-Ar mice compared to vehicle treatment while only male Thy1-Ar mice showed an increase in HVA levels (Figure 4D). Together, duloxetine induced DA and metabolisms in the PFC and HPC showed sex differences.

In contrast to DA, duloxetine induced a significant elevation of 5-HT in PFC and HPC of both male and female mice regardless genotypes, except in the PFC of male WT mice (Figures 4A–D). It is noticed that female Thy1-Ar mice had less response to duloxetine-induced 5-HT level elevation in the HPC compared to that in female WT mice (Figure 4B). In addition, duloxetine promoted 5-HIAA in both PFC and HPC in female mice while males showed genotype specific effects on 5-HIAA levels in the PFC and HPC (Figures 4A–D). In terms of 5-HT turnover, duloxetine only affected the 5-HT index in the PFC, not in the HPC in both male and female mice as shown in Figures 4A–D.

## Discussion

Although many studies showed sex differences in antidepressants, particularly SNRI in treatment of depressive disorders, the mechanisms underlying the sex-dependency remains unclear. Duloxetine is a SNRI and showed some sex-specific effects on depressive-related conditions as sexual dysfunction, severe pain as well as reproductive impairment. To investigate the molecular mechanism of sex-specific effects of duloxetine, we hypothesized that endogenous estrogen-related regulation of neurotransmitter balancing might play a major role. In the present studies, we used very unique animal models to mimic the endogenous estrogen deficiency or overexpression of endogenous estrogen in the brain to study the sex differences in antidepressants. First, we examined the effect of endogenous estrogen on “depressive-liked behaviors” by FST as previous described (Flores-Serrano et al., 2013; You et al., 2017). Our data showed a significant reduction of immobility time in the FST in the male and female Thy1-Ar mice compared to sex-matched WT mice (Figure 1), suggesting that level of endogenous estrogens plays an essential anti-depressive role regardless of sexes, which were in line with previous studies (Rocha et al., 2005; Martínez-Mota et al., 2008; Brummelte and Galea, 2010). In human studies, women with lower level of endogenous estrogen levels showed higher incidence with depression or negative emotions (Grigoriadis and Kennedy, 2002), while ovariectomized animals showed decreased swimming frequency compared to age-matched normal groups (Vega Rivera et al., 2016). However, studies of depressive males showed controversial results on sex hormone dependency. A study showed that castrated male mice had longer immobility time and testosterone administration can reverse the depressive-like behavior (Bernardi et al., 1989), while another research showed no changes of immobility in castrated males regardless testosterone supplementation (Martínez-Mota and Fernández-Guasti, 2004). Our previous studies as well as a recent publication showed an elevated testosterone level in male Ar^+/−^ mice, not in females compared to male WT mice (McAllister et al., 2010; Amano et al., 2017). Therefore, an increased immobile time found in female Ar^+/−^ mice, not in males, suggested that the depression-like behaviors in Ar^+/−^ mice is mainly caused by lacking of estrogen, and testosterone in males might play a role in anti-depressive behaviors (Dalla et al., 2005; Solomon et al., 2009). The testosterone modulated 5-HT2A receptor in males is associated its anti-depressive action (Sumner and Fink, 1998).

Second, we examined the anti-depressive effect of duloxetine in the Ar^+/−^, Thy1-Ar and WT mice by FST. Compared to vehicle controls, duloxetine showed a significant anti-depressant-like effect in both male and female mice regardless of genotypes (Figure 1). Compared to WT mice, duloxetine induced less immobile time in male Ar^+/−^ mice, not in female Ar^+/−^ mice than that in sex-matched WT. Duloxetine made similar anti-depressive effects on FST in female WT, Ar^+/−^ and Thy-Ar mice. Our results suggested that the antidepressant action of duloxetine may not depend on endogenous estrogen, but more likely relies on endogenous testosterone in male mice as elevated testosterone level was only happened in male Ar^+/−^ mice. It is well documented that most antidepressant drugs are associated with altering testosterone levels while there is a correlation between low testosterone and depression (Aguirre, 1999; Margolese, 2000; Bonilla-Jaime et al., 2003; Shebak and Varma, 2014). Recently, a study showed that antidepressants can increase salivary testosterone level in both male and female depression patients who had lower level of testosterone prior treatment (Giltay et al., 2012). The possible interaction between duloxetine and testosterone levels in males was further supported by a study which showed that orchiectomy in rats blocked the anti-depressive effect of desipramine, a NE reuptake inhibitor for depression treatment, and administration of testosterone can be restored the desiprimine’s anti-depressive action (Martínez-Mota and Fernández-Guasti, 2004). These data suggest that the male-favored anti-depressive effect of duloxetine in our behavioral test might be related to the testosterone-related regulation of both 5-HT and NE reuptake inhibition.

Spontaneous locomotor activity of endogenous estrogen or duloxetine was measured to avoid any false-positive stimulatory effect in FST of both female and male mice. Our data showed that both Ar^+/−^ and Thy1-Ar mice administered by vehicle or duloxetine exhibited similar levels of motor activity compared to sex-matched WT mice, respectively. So, the immobility time in FST were not a result of the psychostimulant effects of endogenous estrogen or duloxetine. Such results were similar to previous studies (Dalla et al., 2004, 2005; Solomon et al., 2009; Singh and Singh, 2015).

It is known that the dysfunctions of PFC and HPC play critical roles in development of depression (Berton and Nestler, 2006; Li et al., 2010; Autry et al., 2011), while depressive behavioral changes, such as immobility in the FST is associated with imbalanced DA and 5-HT systems (Liprando et al., 2004). To further understand the involvement of endogenous estrogen in regulation of neurotransmitters, we examined the DA and 5-HT metabolisms in the PFC and HPC of WT, Ar^+/−^ and Thy1-Ar male and female mice. As shown in Figure 3, in the PFC, overexpression of endogenous brain estrogens decreased DOPAC in the PFC of female Thy1-Ar mice compared to female WT, while no significant changes of DA or 5-HT levels in the PFC were noticed. Lacking of endogenous estrogen elevated 5-HIAA level in the PFC of female Ar^+/−^ mice compared to the female WT mice. In the HPC, a great elevation of 5-HT level was found in female Thy1-Ar mice compared to female WT. Although estrogen serves as an antidepressant have been reported by previous publications (Ahokas et al., 2000; Kiss et al., 2012; Kaur et al., 2015), our study first time demonstrated an endogenous estrogen dependent regulation of DA and 5-HT metabolisms in the PFC and HPC. To investigate the estrogen-related DA and 5-HT turnover, we compared DA index and 5-HT index between female Ar^+/−^ or Thy1-Ar mice and female WT mice and found that lacking of endogenous estrogen increased 5-HT index in the PFC and overexpression of brain estrogen reduced 5-HT index in the HPC (Figures 3A,B). Our data also provided a neurochemistry evidence of the linkage between estrogen and depressive behaviors as our behavioral data showed that mice overexpression of brain estrogen reduced immobility while mice were lacking of endogenous estrogen increased immobility in FST compared to WT mice. Our data confirmed that increase of endogenous estrogen plays antidepressant-like roles in animal behaviors and vice versa.

In males, our data showed an elevation of DA and 5-HT levels in the HPC and reduction of 5-HT index in both PFC and HPC of Thy1-Ar mice only (Figures 3C,D). No significant changes of DA or 5-HT systems in male Ar^+/−^ mice compared to male WT mice were found in any of the two brain regions. Interestingly, we found the similar reduction of 5-HT index in the HPC of male and female Thy1-Ar mice. In combining our behavior data which showed reduction of immobility in male and female Thy1-Ar mice, our study suggest that endogenous estrogen-induced anti-depressive effects might be mediated through regulation of 5-HT turnover in the HPC mainly, regardless sex. However, we found an increase of DA level in the HPC of male Thy1-Ar mice, not in females. Whether such a difference of DA level between males and females is responsible for the sex difference in the depression remains unclear. The antidepressant mechanism of endogenous estrogen in 5-HT turnover might involve an increase tryptophan hydroxylase (TPH, restriction enzyme of 5-HT synthesis) activity (Hill and Needham, 2013), reduction of monoamine oxidase (MAO), a key enzyme in 5-HT degradation (Wu et al., 2009), restored impaired serotonin transporter (SERT) function (Zha et al., 2017), and reduction of brain nitric oxide (NO) level, a modulator for 5-HT (Harkin et al., 2004; Heydarpour et al., 2013). Although estrogen serves as an antidepressant as reported by many studies, the use of estrogen in the treatment of depression was still limited by its side effects, such as mammary cancer (Kubista et al., 2007).

When the DA system was analyzed in female mice, the present findings found that duloxetine promoted DA level and decreased DA index of HPC in WT and Thy1-Ar mice compared to genotype-matched vehicle treated groups. No significant differences between duloxetine treated female mice. For the 5-HT metabolisms, we found that 5-HT and 5-HIAA levels were increased in both PFC and HPC of all female mice except 5-HIAA in the PFC of female Ar^+/−^ mice. As shown in Figure 4A, duloxetine treatment decreased the 5-HT index of PFC in female WT and Thy1-Ar mice, not in female Ar^+/−^ mice. Our data suggest that lacking of endogenous estrogen attenuated the response to duloxetine while overexpression of brain estrogen did not change the duloxetine efficacy in females compared to vehicle treatment. Dopaminergic and serotonergic receptors are target of duloxetine and can be modulated by estrogen (Dhir and Kulkarni, 2008). Several animal and human studies demonstrated that estrogen increases the efficacy of DA system in females (Kritzer and Creutz, 2008; Jacobs and D’Esposito, 2011; Rey et al., 2014). In addition to the effect of estrogen on modulation of serotonergic function by the regulation of TPH and the expression of 5-HT transporters and receptors (Sánchez et al., 2011; Yamaguchi et al., 2016), estrogen might also regulate duloxetine metabolism. For instance, duloxetine can be metabolized by cytochrome P450 (CYP) 2D6 and 1A2 (Fric et al., 2008) in the liver. Studies found that estrogen might negatively correlated with CYP 2D6 and CYP 1A2 activity (Gex-Fabry et al., 1990; Ereshefsky et al., 1991; Relling et al., 1992; Parkinson et al., 2004; Bartkowiak-Wieczorek et al., 2015; Xie et al., 2017), which might explain that attenuation of endogenous estrogen attenuated the response to duloxetine noted in Ar^+/−^ female mice. In addition, compared to duloxetine-treated female WT, female Thy1-Ar mice showed less elevation of 5-HT and 5-HIAA levels in the HPC (Figure 4B). The differences of 5-HT metabolism between female Thy1-Ar and sex-matched WT could be due to the higher basal level of 5-HT and 5-HIAA in Thy1-Ar mice as shown in Figure 4B.

While male mice showed similar changes of 5-HT levels and 5-HT index treated by duloxetine in the PFC and HPC as found in females. Interestingly, duloxetine caused different effects on DA metabolisms in males. Compared to vehicle groups, both Ar^+/−^ and Thy1-Ar male mice showed an elevation of DA levels induced by duloxetine in the PFC and HPC, while male Thy1-Ar mice showed higher level of DA than that in male Ar^+/−^ mice in the PFC (Figures 4C,D). While an increase of DA level might be a common feature of antidepressants (D’Aquila et al., 2000), it is unclear why male Ar^+/−^ and Thy1-Ar both had similar response to duloxetine-induced DA elevation. One of the possibilities is testosterone. It is know that testosterone primarily acts through its 5α-reduced product dihydrotestosterone and the aromatase-derived estradiol in corticolimbic neural circuits, such as the PFC, the amygdala and the HPC (Roselli et al., 2009; McHenry et al., 2014; Puralewski et al., 2016). In intact male rodents, testosterone increased DA and 5-HT release in the neostriatum and nucleus accumbens (de Souza Silva et al., 2009). Compared with males, DA release of females were more sensitive to estrogen (Becker, 1999; Barker and Galea, 2008), while males were more sensitive to dopamine receptor antagonist than females (Arenas et al., 1999; Parra et al., 1999). As our previous studies showed that male Ar^+/−^ mice have an elevation of endogenous testosterone level which might synchronize the duloxetine-induced increase in DA level as demonstrated in our data. Instead of aromatase deficiency globally as the Ar^+/−^ mice, the Thy1-Ar mice promoted neuronal aromatase which produce more estrogen locally. It is possible that the changes of testosterone level in male Thy1-Ar mice is limited compared to the male Ar^+/−^ mice. Therefore, the effect of estrogen on duloxetine-induced DA level might override the effect of testosterone in male Thy1-Ar mice. In concert with our hypothesis, other studies showed that estrogen enhanced the Venlafaxine (another SNRI) anti-depressive effect in male mice (Dhir and Kulkarni, 2008).

According to the current knowledge, PFC and HPC are commonly associated with depression, as well as in antidepressant response (Bredy et al., 2003; Cerqueira et al., 2007; Drevets et al., 2008; Bagot et al., 2009; Bessa et al., 2009; Monroy et al., 2010; Sequeira-Cordero et al., 2013). However, some study showed that antidepressant intervention had a greater effect on 5-HT level of HPC compared to that in the PFC (Bekris et al., 2005; Pitychoutis et al., 2012), while other study showed a better linkage between the antidepressant-treated behavioral outcomes and the 5-HT system in the PFC rather than that in the HPC (Mikail et al., 2012). Such controversial findings might be related to specific antidepressant or specific interaction between antidepressant and neurotransmitters. For example, the 5-HT system in the PFC seemed to be associated with reward behavior and positive affections (Varea et al., 2007), while the roles of 5-HT and DA in HPC functions may involve emotional alterations and anti-depression (Robertson et al., 2005; Boldrini et al., 2012; Jiang et al., 2017).

Recent findings showed that cAMP response element-binding protein (CREB)—brain derived neurotrophic factor (BDNF) pathway in the HPC was closely related in depression and antidepressants (Rojas et al., 2011; Breuillaud et al., 2012; Takano et al., 2012). The CREB is an upstream transcription factor for BDNF expression, and CREB could also be activated by BDNF. It was well documented that CREB and BDNF has pivotal roles in both pathophysiology and treatment of depression (Castrén and Rantamäki, 2010; Guo et al., 2014; Hashimoto, 2015). Elevated CREB level was observed in the HPC of mice treated with venlafaxine (another SNRI) than that in the control mice (Shen et al., 2017). There is a possibility that duloxetine plays antidepressant role by increasing the level of BDNF in PFC and HPC (Prickaerts et al., 2012).

The receptors of 5-HT genes have been targeted in models of depression. Male and female 5-HT1A receptors knockout mice showed reduced immobility time in FST, but only male mice exhibit decreased preference for sucrose (Jones and Lucki, 2005; Castagné et al., 2011; Alexander et al., 2013); while female mice lacking 5-HT1B receptors showed more depressive behavior than genotype-matched male mice (Jones and Lucki, 2005). In contrast, female but not male 5-HT3 knockouts show depressive behavior in FST (Bhatnagar et al., 2004). BDNF knockout mice are used as an another model of depression and show marked sex differences. Female rats but not male rats exhibited depression-related behaviors (Monteggia et al., 2007; Autry et al., 2009). Depressive behavior was founded in male mice lacking the forebrain type II glucocorticoid receptor NR3C1(FBGRKO) but not in female mice (Albelda and Joel, 2012; Solomon et al., 2012).

Sex differences had also been observed in genetic rodent models of depression. For instance, Flinders Sensitive Line (FSL) rats, a genetic model of depression, both male and female of them showed depressive symptoms (Overstreet, 1986). And male FSL rats exhibited more depression-like behavior than female (Kokras et al., 2009). The Wistar Kyoto (WKY) rats were accepted by another genetic depressive model. WKY rats can be divided into two inbred sub-strains, one with more immobile (WMI) and another with less immobile (WLI) rats according to immobility time in the FST. Female WMIs showed depressive-like behaviors only in adulthood. But WMI males displayed such behaviors from adolescence to adulthood. The sex difference was attributed to the gene expression differences in HPC (Mehta et al., 2013).

Together, our data demonstrated that duloxetine affected 5-HT metabolism more than DA system in females, while both DA and 5-HT were significantly affected by duloxetine treatment in male mice regardless of which brain regions.

## Conclusion

As our data showed, increase of endogenous estrogen produced an antidepressant-like effect in FST of female and male mice by regulating DA and 5-HT. Lacking of endogenous estrogen reduced antidepressive effect of duloxetine in females only. The sex difference of duloxetine in FST is mainly regulated by 5-HT and DA system of PFC and HPC, such as the effect of duloxetine did not depend on the level of endogenous estrogen in females, while endogenous testosterone can enhance the antidepressant effect of duloxetine in males. Our studies suggested that the level of estrogen or testosterone in patient with MDD should be measured and considered prior administration by antidepressants, in order to achieve optimal efficacy of treatment.

## Author Contributions

YX designed, conducted and analyzed the experiments. LM and WJ contributed mice and designed the experiments. GW and RL designed and conceived the experiments. All authors discussed the results and contributed to writing the manuscript.

## Conflict of Interest Statement

The authors declare that the research was conducted in the absence of any commercial or financial relationships that could be construed as a potential conflict of interest.
